# Highly Sensitive Paper-Based Force Sensors with Natural Micro-Nanostructure Sensitive Element

**DOI:** 10.3390/nano14040358

**Published:** 2024-02-14

**Authors:** Haozhe Zhang, Yuyu Ren, Junwen Zhu, Yanshen Jia, Qiang Liu, Xing Yang

**Affiliations:** 1 Department of Precision Instrument, Tsinghua University, Beijing 100084, China; zhanghz18@tsinghua.org.cn (H.Z.); zhujw21@mails.tsinghua.edu.cn (J.Z.); jys20@tsinghua.org.cn (Y.J.); qiangliu@tsinghua.edu.cn (Q.L.); 2 Key Laboratory of Photonic Control Technology (Tsinghua University), Ministry of Education, Beijing 100084, China; 3 State Key Laboratory of Precision Space–Time Information Sensing Technology, Beijing 100084, China; 4 School of Materials Science and Engineering, University of Science and Technology Beijing, Beijing 100083, China; 13519110246@163.com

**Keywords:** paper-based sensor, micro-nanostructure, sensitive element, paper material, high sensitivity, force sensor, pressure sensor, strain sensor

## Abstract

Flexible paper-based force sensors have garnered significant attention for their important potential applications in healthcare wearables, portable electronics, etc. However, most studies have only used paper as the flexible substrate for sensors, not fully exploiting the potential of paper’s micro-nanostructure for sensing. This article proposes a novel approach where paper serves both as the sensitive element and the flexible substrate of force sensors. Under external mechanical forces, the micro-nanostructure of the conductive-treated paper will change, leading to significant changes in the related electrical output and thus enabling sensing. To demonstrate the feasibility and universality of this new method, the article takes paper-based capacitive pressure sensors and paper-based resistive strain sensors as examples, detailing their fabrication processes, constructing sensing principle models based on the micro-nanostructure of paper materials, and testing their main sensing performance. For the capacitive paper-based pressure sensor, it achieves a high sensitivity of 1.623 kPa^−1^, a fast response time of 240 ms, and a minimum pressure resolution of 4.1 Pa. As for the resistive paper-based strain sensor, it achieves a high sensitivity of 72 and a fast response time of 300 ms. The proposed new method offers advantages such as high sensitivity, simplicity in the fabrication process, environmental friendliness, and cost-effectiveness, providing new insights into the research of flexible force sensors.

## 1. Introduction

Flexible force sensors have important potential applications in fields such as healthcare wearables, artificial intelligence, the Internet of Things, portable electronics, and interactive robots [1,2,3,4,5,6,7], and have become one of the hot research topics currently. Although the sensing principles and materials used in flexible force sensors vary across studies, they typically include two key components: a sensitive element and a flexible substrate [8,9,10]. The flexible force sensors have many advantages, such as high sensitivity, small size, and good flexibility, but there are still some challenges with these two components: (1) The sensitive element, being the most crucial part of flexible force sensors, often employs novel nanomaterials like carbon nanotubes, graphene, metal oxide semiconductors, nanowires, and nanoparticles [11,12,13,14,15,16,17] to achieve high sensitivity. However, the fabrication process of sensors using these novel nanomaterials is usually complex and lacks consistency in mass production, limiting their large-scale manufacturing in practical applications. Additionally, the high cost of these novel nanomaterials makes the production of flexible force sensors relatively expensive. (2) For the flexible substrate of the flexible force sensors, polymer materials like polydimethylsiloxane (PDMS), polyurethane (PU), polyimide (PI), and polyethylene terephthalate (PET) are commonly used [18,19,20,21]. These polymer materials offer good flexibility, high heat resistance, and high chemical resistance [10]. However, their non-biodegradability can contribute to environmental pollution, and the integration of the flexible substrates with the sensitive elements composed of novel nanomaterials can complicate the fabrication process of the sensors.

Therefore, in response to these challenges, there is an urgent need to develop flexible force sensors that are simple in fabrication, low-cost, eco-friendly, and possess excellent sensing characteristics such as high sensitivity, wide measurement range, and fast response time. In this article, we focus on ordinary paper as the material of the sensor. Paper, invented thousands of years ago, has long been used for writing, recording, printing, painting, and packaging [22]. Paper has the following significant characteristics that make it suitable as both the sensitive element and flexible substrate for flexible force sensors: (1) The paper material itself has natural micro-nanostructures, such as paper composed of crisscrossing micro-nanofibers with obvious pore structure, and after conductive treatment of paper fibers, a large number of micro-cracks can be produced, which can be used to improve the sensitivity of sensing. Based on these natural micro-nanostructures, the relevant electrical output (such as capacitance, resistance, inductance, etc.) of the paper after conductive treatment will change with mechanical input, thus enabling high-sensitivity sensing. Therefore, paper itself can serve as the sensitive element of flexible force sensors, eliminating the need for additional nanomaterials like carbon nanotubes or graphene, thus simplifying the fabrication process and reducing costs. (2) As a flexible material, paper can undergo deformation operations, such as bending, folding, and twisting with relative ease, thereby ensuring good conformity to the surface of the object being measured. Additionally, it is eco-friendly, inexpensive, easy to obtain, and lightweight, making it highly suitable for serving as both the flexible substrate and the sensing element of a sensor. (3) The conductive treatment of paper is also relatively straightforward. Simple techniques like writing, printing, pasting, spraying, and sputtering can be used to create conductive layers, encapsulation layers, leads, and variable units on the paper-based sensor’s surface. The sensor’s shape can also be tailored through simple techniques like folding, cutting, and laser cutting, enhancing its sensitivity and measurement range. However, achieving these with simple processing techniques is often challenging when using polymer materials for sensor fabrication. In summary, the three characteristics of paper mentioned above allow it to serve simultaneously as a sensitive element and flexible substrate of the force sensor, reducing the steps needed to integrate these components and enabling further processing using simple techniques. Therefore, using paper to make sensors offers great advantages in terms of simplicity, cost-efficiency, and eco-friendliness.

In recent years, paper materials have been successfully applied in various fields such as supercapacitors, microfluidics, chemical analysis, temperature measurement, and humidity detection [23,24,25,26]. Simultaneously, there have also been studies on utilizing paper materials to fabricate flexible force sensors, the main principle and methodology involve using paper materials as the flexible substrate of the sensor and assembling or fabricating materials, such as carbon nanotubes, graphene, carbon black, graphite, metal oxide semiconductors, Ag/Au nanowires, and ZnO nanoparticles, on the paper to serve as the sensing element for detecting mechanical signals. For example, Venkatarao Selamneni et al. used a simple, low-cost hydrothermal synthesis method to deposit molybdenum diselenide (MoSe_2_) on cellulose paper, thus creating a flexible piezoresistive pressure and strain sensor with the cellulose paper as the substrate and MoSe_2_ as the sensitive element. The pressure sensor had a sensitivity coefficient close to 0.3 kPa^−1^, and the strain sensor’s sensitivity coefficient was as high as 6.84, maintaining good stability after 500 bending cycles [27]. Ren Tianling et al. created a graphene paper by suspending paper towels in a graphene oxide solution and then thermally reducing it, fabricating a flexible piezoresistive pressure sensor with the paper towel as the substrate and graphene as the sensitive element. This sensor could measure a large pressure range of 0~20 kPa with a high-sensitivity coefficient of 17.2 kPa^−1^ [28]. Lee, Kilsoo et al. drew graphite conductive layers using an 8B pencil on paper, thus making a flexible capacitive pressure sensor with the paper as the substrate and graphite as the sensitive element, which had a sensitivity of 0.69 kPa^−1^, a pressure resolution of 6 Pa, and good stability after 5000 cycles [29]. Hanbin Liu et al. immersed filter paper in a mixture of carbon black and carboxymethyl cellulose (CMC), creating a flexible resistive strain sensor with the filter paper as the substrate and carbon black and CMC as the sensitive element. This sensor had a sensitivity coefficient of 4.3, a response time of 420 ms, and maintained good stability after 1000 stretches [30]. Hemtej Gullapalli et al. used a low-temperature solvent thermal method to grow ZnO nanofiber structures on paper, thus producing a flexible piezoelectric strain sensor with the paper as the substrate and ZnO nanoparticles as the sensitive element, achieving a sensitivity coefficient of 21.12 [31].

**Figure 1:**
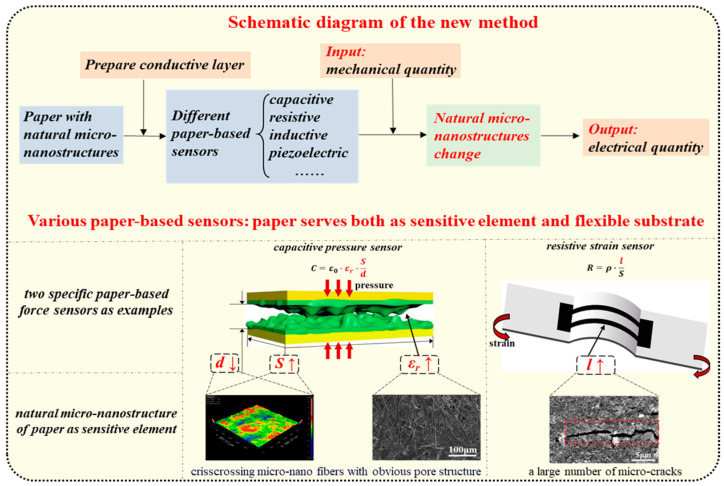
Schematic diagram of the proposed new method and several examples of paper-based force sensors that utilize the natural micro-nanostructure of paper as the sensitive element.

The aforementioned studies demonstrate that using paper as the flexible substrate and fabricating the sensitive element on it can achieve high sensitivity, wide measurement range, good cycling stability, and fast response time. However, these studies only utilized the good flexibility and conformability of the paper, employing it solely as the flexible substrate for sensors. Furthermore, can we also use the natural micro-nanostructure of the paper itself as the sensor’s sensitive element? Therefore, as illustrated in Figure 1, this article creatively proposes a new method where paper serves both as the sensitive element and the flexible substrate of flexible force sensors: After conductive treatment, the conductive pathways within the micro-nanostructures of the paper itself undergo changes under the influence of mechanical quantities (such as pressure and strain), leading to changes in its related electrical output (such as capacitance, resistance, and inductance), thereby achieving force sensing. This method simplifies the fabrication process as it does not require the deposition of additional materials on the paper to prepare the sensitive element. Based on this proposed new method, this article presents capacitive pressure sensors and resistive strain sensors as examples, demonstrating the feasibility and universality of this approach. For these two specific sensors, the article describes their fabrication processes, constructs sensing principle models based on the micro-nanostructure of paper materials, and tests their main sensing performance, proving that the flexible force sensors fabricated using this new method are not only simple in the process but also exhibit excellent sensing performance, including high sensitivity, wide measurement range, fast response time, and good cycling stability.

## 2. Experimental Section

### 2.1. Device Fabrication

**Figure 2:**
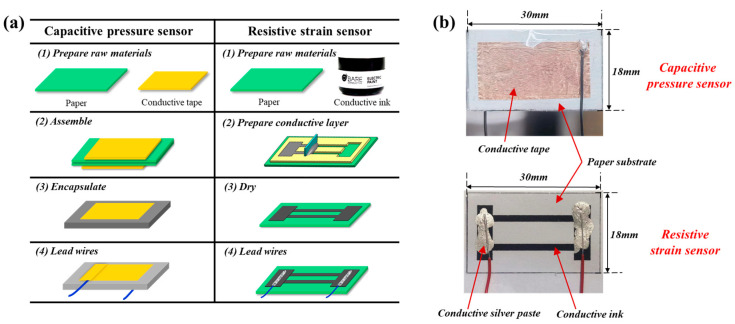
(**a**) Fabrication process of capacitive pressure and resistive strain paper-based sensors; (**b**) photographs of the two types of sensors with dimensional specifications.

The capacitive paper-based pressure sensor consists of four parts: a flexible substrate, a sensitive element, a conductive layer, and an encapsulating layer. Specifically, paper serves both as the flexible substrate and the sensitive element, conductive tape as the conductive layer, and BOPP tape as the encapsulating layer. The fabrication process is shown on the left side of Figure 2a. First, cut the paper into an 18 × 30 mm^2^ rectangle and cut the conductive tape into a slightly smaller rectangular thin sheet, then adhere the conductive tape to the smoother side of the paper. For a capacitive sensor made of two layers of paper, assemble the two pieces of paper face to face on their rougher sides, as shown in step (2). Then, encapsulate the sensor with BOPP tape to fix the relative positions of the two layers of paper, protecting the sensor for stable operation during testing. Finally, lead wires on the upper and lower surfaces of the sensor to obtain the sensor’s output capacitance signal.

The resistive paper-based strain sensor consists of three parts: a flexible substrate, a sensitive element, and a conductive layer. To be specific, paper serves as the flexible substrate, the natural micro-nanofibers of the paper infiltrated with conductive ink act as the sensitive element, with conductive silver paste and conductive ink as the conductive layer. The fabrication process is shown on the right side of Figure 2a. First, prepare a patterned template as shown in step (2), cover it on a specific area of the paper, and evenly spread conductive ink (Electric Paint 50 mL, Bare Conductive, London, UK) on the template’s hollowed-out parts using a squeegee. Remove the template to achieve the graphic transfer of the conductive ink on the paper. Then, leave the ink-coated paper in the air for at least 12 h, allowing the solvent and moisture in the conductive ink to fully evaporate. Finally, lead wires along the length direction of the electrodes on both sides of the conductive ink, as shown in step (4). As paper cannot withstand high temperatures, conductive silver paste (#5001-AB, SPI Supplies, West Chester, PA, USA) that can connect wires with conductive ink at room temperature is used to obtain the sensor’s output resistance signal. This sensor can also have an encapsulating layer added as needed for practical applications, though it is not shown in the figure.

Figure 2b illustrates the physical photographs and dimensional specifications of the two types of sensors. It is important to note that the dimensions shown in the figure are merely an example of sensor fabrication and can be flexibly adjusted according to specific application requirements.

### 2.2. Characterization and Measurement

For characterization, a high-resolution scanning electron microscopy (Model 450, NOVA NANOSEM, New York, NY, USA) is used to characterize the surface morphology of the sensors; a 3D white light interferometric surface profilometer (Model NEXVIEW, ZYGO, Middlefield, CT, USA) is used to characterize the surface roughness of the sensors; an automatic specific surface and porosity analyzer (Model 3-Flex, MICROMERITIES, Norcross, GA, USA) is used to conduct BET tests for different paper materials.

In sensor testing, for the capacitive paper-based pressure sensor, a custom-built pressurizing device combined with an impedance meter (Model WK6500B, WAYNE KERR, Bognor Regis, UK) is used for pressure loading and signal acquisition. For the resistive paper-based strain sensor, a tensile test platform (Model Z825B, THORLABS, Londonderry, NH, USA) is used to bend and compress the sensor, and a precision picoammeter (Model 6487, KEITHLEY, Cleveland, OH, USA) is used for real-time collection of output electrical signals.

## 3. Results and Discussion

### 3.1. Capacitive Paper-Based Pressure Sensor

#### 3.1.1. Micro-Nanostructure Characterization and Sensing Principle

**Figure 3:**
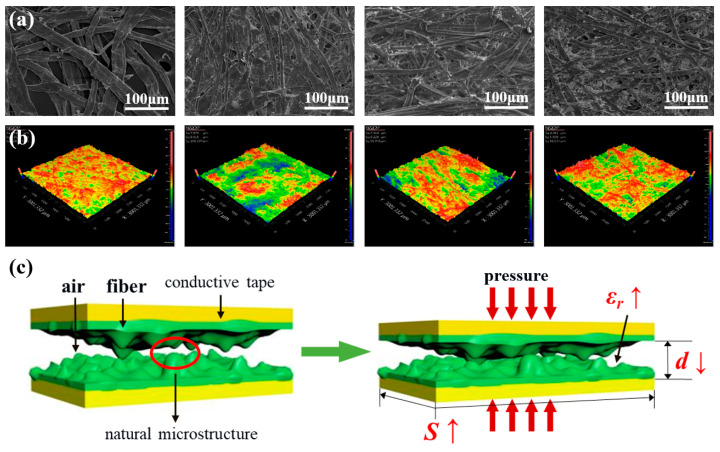
(**a**) Surface morphology images of lens paper, rice paper, kraft paper, and printing paper; (**b**) surface roughness images of lens paper, rice paper, kraft paper, and printing paper; (**c**) theoretical model of the sensing principle for a capacitive paper-based pressure sensor.

To investigate how the inherent micro-nanostructure of paper affects the sensitivity of paper-based capacitive pressure sensors, this study selected four typical paper materials with different microstructures, namely lens paper, rice paper, kraft paper, and printing paper. These materials were characterized using a scanning electron microscope and a 3D white light interferometer for their surface morphology and surface roughness, respectively. Figure 3a shows the surface morphology images of the four types of paper. It can be observed that all four types of paper exhibit some common microscopic features, including crisscrossing micro-nanofibers with obvious pore structures. However, they also exhibit differences in fiber density and porosity rate ($porosity\;rate=\frac{void\;volume}{total\;volume}$), with the porosity rate decreasing in the order of lens paper, rice paper, kraft paper, and printing paper. Lens paper and rice paper have a significantly higher porosity rate compared to kraft paper and printing paper. This conclusion can also be quantitatively supported by the results of the BET test, as shown in Supplementary Figure S1. Figure 3b shows the surface roughness images of the four types of paper, where different colors represent different heights on the paper surface, with blue and red indicating lower and higher areas, respectively. The measured surface roughness of lens paper, rice paper, kraft paper, and printing paper are 6.599, 7.670, 7.319, and 4.361 μm, respectively, indicating that rice paper, kraft paper, and lens paper have a much higher surface roughness than that of printing paper.

The sensitivity calculation formula for the capacitive pressure sensor [32] is as follows:

(1) $$S=\frac{∆C/C_{0}}{∆P}$$

where $C_{0}$ represents the capacitance value of the sensor when it is not subjected to pressure; ∆C represents the change in capacitance when the sensor is under pressure; ∆P represents the pressure value exerted on the surface of the sensor. Combining the surface morphology and roughness of these four types of paper, the theoretical model constructed for this capacitive pressure sensor suggests that its sensitivity is closely related to the porosity rate and surface roughness of the paper material. The higher the porosity rate and surface roughness, the higher the sensitivity of the sensor. As shown in Figure 3c, this sensor can be considered as a parallel-plate capacitor, where the paper acts as the dielectric layer with a thickness d, an area of S, and a dielectric constant of $\varepsilon_{r}$. The paper can be viewed as a mixture of cellulose and air in the pores, with the dielectric constant of cellulose being higher than that of air. According to the parallel-plate capacitor calculation formula,

(2) $$C=\varepsilon_{0}\varepsilon_{r}\cdot \frac{S}{d}$$

when pressure is applied, the thickness d of the dielectric layer decreases, the area S increases, and the dielectric constant $\varepsilon_{r}$ increases, leading to an increase in the output capacitance C. When the same pressure is applied and the surface roughness of the paper is the same, sensors with a higher porosity rate on their surfaces will expel more air from the dielectric layer, resulting in a more significant increase in the dielectric constant $\varepsilon_{r}$, and hence higher sensitivity. Similarly, when the same pressure is applied and the porosity rate of the paper is the same, sensors with a rougher paper surface will lead to a more noticeable decrease in the thickness d of the dielectric layer, leading to higher sensitivity. In conclusion, the higher the porosity rate and surface roughness of the paper material, the higher the sensitivity of the capacitive pressure sensor.

#### 3.1.2. Sensor Performance Testing

**Figure 4:**
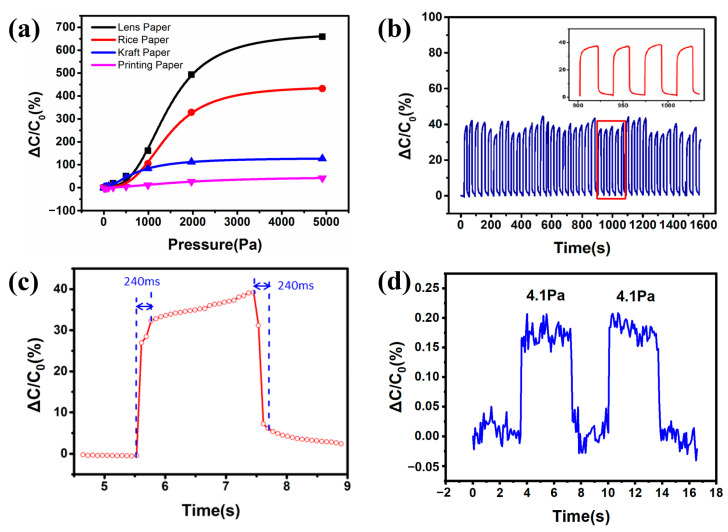
(**a**) Comparison of sensitivity curves of capacitive pressure sensors made from four types of paper materials; (**b**) cycling stability test of the rice paper-based capacitive pressure sensor under 60 Pa pressure; (**c**) response time curve of the rice paper-based capacitive pressure sensor; (**d**) minimum pressure resolution curve of the rice paper-based capacitive pressure sensor.

To experimentally validate the theoretical model based on the micro-nanostructure of paper, capacitive pressure sensors based on lens paper, rice paper, kraft paper, and printing paper were fabricated following the process outlined in Figure 2a, and sensitivity tests were conducted on sensors made from these four different paper materials. The relationship between the capacitance change percentage and applied pressure for each sensor is shown in Figure 4a. The sensitivities of these four types of paper-based capacitive pressure sensors in the 0~2 kPa pressure range are 2.385, 1.623, 0.636, and 0.145 kPa^−1^, respectively, and in the larger pressure range of 2~5 kPa, the sensitivities are 0.563, 0.308, 0.053, and 0.045 kPa^−1^, respectively. Among them, the lens paper-based and rice paper-based sensors exhibit higher sensitivity, significantly surpassing the sensitivity of sensors based on kraft paper and printing paper. According to the theoretical model, this is due to the higher porosity rate and surface roughness of lens paper and rice paper, enabling higher sensitivity pressure detection over a wide pressure range.

Additionally, aside from sensitivity, the repeatability of the sensors after multiple pressure cycles is also an important criterion for selecting paper materials. Multiple sensitivity tests were performed on lens paper-based and rice paper-based capacitive pressure sensors, with their curves shown in Supplementary Figure S2. It is observed that although the lens paper-based sensor has a slightly higher sensitivity than that of the rice paper-based sensor, its repeatability after multiple pressure cycles is noticeably worse. This is because the fibers of lens paper are sparser and individual fibers are larger, making them more prone to damage during continuous loading and unloading processes, thus leading to changes in capacitance output under the same pressure, resulting in poorer repeatability. Therefore, rice paper was ultimately selected to fabricate capacitive pressure sensors, and further studies were conducted on the performance of rice paper-based capacitive pressure sensors.

Figure 4b shows the capacitance relative change of the sensor during dozens of loading cycles at a pressure of 60Pa, it can be seen that the output capacitance remains relatively stable, indicating the sensor’s high stability in pressure detection over long periods and multiple cycles. Figure 4c shows that the response time of the rice paper-based capacitive pressure sensor during loading and unloading is roughly 240 ms, enabling rapid detection of pressure signals. Figure 4d demonstrates that the sensor can discern a minimum pressure of 4.1 Pa, allowing for precise measurement of minute pressure.

In summary, suitable paper materials can be selected to prepare paper-based capacitive pressure sensors with specific performance requirements. These sensors exhibit high sensitivity, high stability, fast response time, and high pressure resolution. Utilizing the micro-nanostructure of paper itself, the paper material can be simultaneously used as a sensitive element and flexible substrate of the sensor, which provides advantages in terms of simple fabrication, low cost, and eco-friendliness.

### 3.2. Resistive Paper-Based Strain Sensor

#### 3.2.1. Micro-Nanostructure Characterization and Sensing Principle

From the characterization of the surface morphology and roughness of the four types of paper materials in Section 3.1.1, it is known that among these four materials, printing paper has a relatively smooth and flat surface with low roughness, which is more conducive to the uniform coating of conductive inks. Additionally, the fibers on the surface of printing paper are more densely packed and uniformly sized, facilitating a tight bond between the conductive ink and paper fibers, thus providing stable electrical signal transmission even after multiple deformations. Therefore, printing paper was chosen for fabricating the resistive paper-based strain sensor.

**Figure 5:**
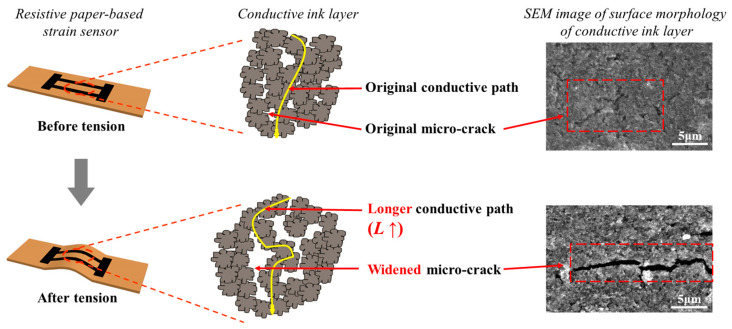
Micro-nanostructure characterization and theoretical model of the sensing principle for resistive paper-based strain sensor.

The theoretical model of its sensing principle is shown in Figure 5. The conductive ink, tightly bound to the paper fibers, is formed by the accumulation of tiny conductive particles, resulting in a rough surface with a certain number of micro-cracks after drying. When the sensor undergoes deformation due to bending, some cracks widen and new cracks form, lengthening the electron’s conductive path. According to the resistance calculation formula,

(3) $$R=\rho \cdot \frac{L}{S}$$

the increase in conductive path L causes an increase in the resistance R of the sensor’s sensitive element. The sensitivity calculation formula for the resistive strain sensor is as follows:

(4) $$GF=\frac{∆R/R_{0}}{\varepsilon }$$

where $R_{0}$ represents the resistance value of the sensor when it is not subjected to tension or compression; ∆R represents the change in resistance when the sensor is under tension or compression; ε represents the magnitude of the sensor’s strain, which is the ratio of the change in length of the sensor due to tension or compression to the original length of the sensor. The diagram for the specific calculation of strain ε can be found in Supplementary Figure S3.

Subsequent experimental results demonstrate that even under small strains, the sensor exhibits a relatively large change in resistance resulting from the widening of micro-cracks and the formation of new cracks, thereby endowing the sensor with high sensitivity.

#### 3.2.2. Sensor Performance Testing

**Figure 6:**
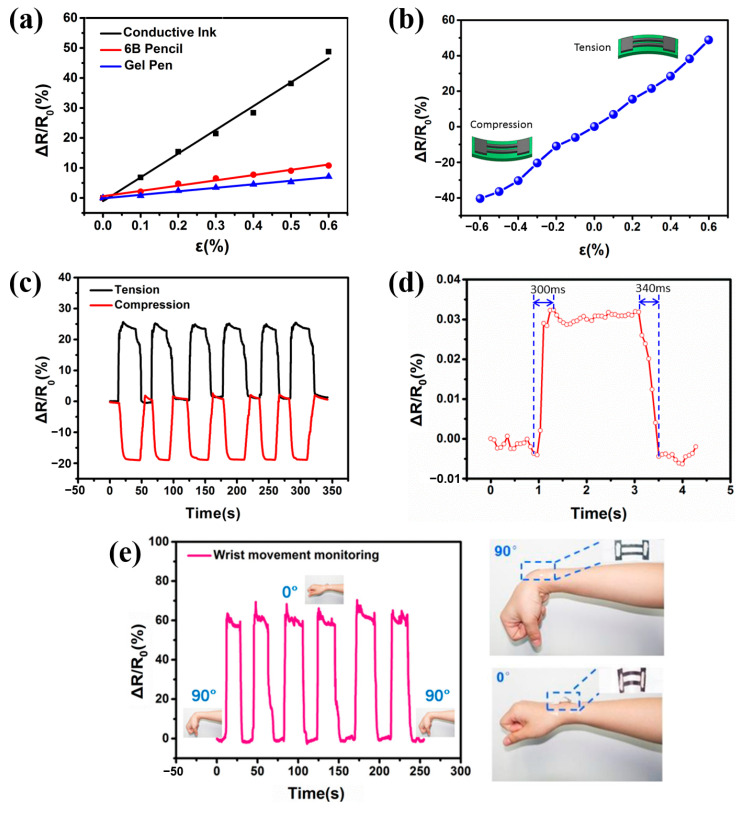
(**a**) Comparison of sensitivity curves for resistive strain sensors with sensitive elements prepared using conductive ink, 6B pencil, and gel pen; (**b**) sensitivity curves of the resistive strain sensor under tension and compression; (**c**) dynamic testing curves of the resistive strain sensor under tension and compression at 0~0.3% strain; (**d**) response time of the resistive strain sensor during strain loading and unloading; (**e**) real-time monitoring curve of wrist motion posture by the resistive paper-based strain sensor.

The sensitive elements can not only be prepared on the surface of paper by coating conductive ink but can also be made by writing with a 6B pencil or a gel pen. As shown in Figure 6a, the sensitivity of resistive strain sensors fabricated using these three methods was measured. The result shows that the sensor fabricated with conductive ink exhibits the largest resistance change with increasing strain, indicating the highest sensitivity. Therefore, subsequent experiments used resistive strain sensors with conductive ink as the sensitive element for further performance testing.

Figure 6b shows the curve of the sensor’s relative resistance change versus strain under compression and tension, shown in the lower left and upper right of the figure, respectively. It is evident that with increasing compressive or tensile strain, the sensor’s resistance change roughly linearly correlates with the strain change, and the sensor’s sensitivity coefficient is calculated to be 72, enabling a high-sensitivity detection of strain.

Cyclical compressive and tensile strains (0~0.3%) were applied to the sensor, and its resistance change curve is shown in Figure 6c. It can be seen that the sensor maintains good stability in its resistance output during continuous loading and unloading. Microscopically, this is due to the dense and uniform fiber structure of the printing paper, ensuring a tight bond between the conductive ink and paper fibers. Even after multiple deformations, the conductive ink remains firmly adhered to the paper surface, enabling reliable transmission of electrical signals.

Figure 6d shows the response times of the sensor during loading and unloading are 300 ms and 340 ms, respectively. Microscopically, this is because when strain is applied, the widening of micro-cracks and the formation of new cracks take time. When the strain is unloaded, it also takes some time for micro-cracks to recover to their initial state, thereby causing a lag in the electrical signal.

Figure 6e displays the real-time monitoring curve of wrist motion posture using the sensor. The sensor is attached to the back of the wrist. When the hand bends downwards at a 90° angle to the forearm, the sensor experiences no strain and remains in a natural state. When the hand is raised in line with the forearm, the sensor bends upwards, indicating a state of tension. As the angle between the hand and forearm changes, the sensor’s resistance value changes in real time. After multiple bending and releasing actions of the wrist, the sensor continues to output a stable resistance value, demonstrating good stability and potential for applications in wearable devices.

In summary, the sensor exhibits high sensitivity, high stability, and fast response time. In terms of its sensing principle, it utilizes the micro-cracks formed by the tight combination of conductive materials with paper fibers for strain sensing. Therefore, paper materials can serve as both sensitive elements and flexible substrates of the sensor, offering advantages in terms of simple fabrication, low cost, and eco-friendliness, providing a new approach for the research of resistive strain sensors.

## 4. Conclusions

This article proposes a novel method of using paper as both the sensitive element and the flexible substrate for flexible force sensors. This method fully exploits the inherent micro-nanostructure of paper as the sensitive element, which consists of interlaced micro-nanofibers with significant pore structures, and after the conductive treatment of paper fibers, numerous micro-cracks that increase sensitivity can be produced. These micro-nanostructures of paper will change under external mechanical forces, leading to alterations in related electrical outputs, thereby achieving force sensing. This method simplifies the fabrication process, as it eliminates the need for depositing additional materials on the paper surface to create the sensitive element. To demonstrate the feasibility and universality of this method, this article focused on two types of commonly used sensors, capacitive paper-based pressure sensors and resistive paper-based strain sensors, as examples. For each type of sensor, the sensing principle and fabrication process were studied, a sensing principle model based on the paper’s micro-nanostructure was constructed, and various typical performance indicators were tested. For the paper-based capacitive pressure sensor, the fabrication process is simple, involving only cutting, pasting, encapsulating, and wiring. In terms of sensing performance, it achieves a high sensitivity of up to 1.623 kPa^−1^ and a fast response time of 240 ms. It can not only detect a wide pressure range of 0~5 kPa but also discern a minimum pressure of 4.1 Pa. The sensor also maintained stable capacitance output over multiple loading cycles. For the paper-based resistive strain sensor, the fabrication process is equally simple, involving only the preparation of patterned templates, coating of conductive materials, drying, and wiring. This sensor exhibits a high sensitivity of 72 and a fast response time of approximately 300 ms. It also proved capable of accurately and stably monitoring human wrist movements in real time. In summary, both types of paper-based force sensors demonstrated possess great sensing performances and simple fabrication processes compared to other studies that utilize cellulosic materials for fabricating flexible force sensors (as shown in Supplementary Tables S1 and S2), confirming the feasibility and universality of using paper as both the sensitive element and flexible substrate for force sensors. This method is promising for providing new insights into the research of flexible force sensors, and the sensors fabricated using this method, characterized by their high sensitivity, simple process, eco-friendliness, and cost-effectiveness, have the potential for application in fields such as healthcare wearables, portable electronics, and artificial intelligence.

## Data Availability

The data presented in this study are available upon request from the corresponding author.

## Supplementary Materials

The following supporting information can be downloaded at: https://www.mdpi.com/article/10.3390/nano14040358/s1. Figure S1. (a) BET experimental apparatus used for testing; (b) comparison of cumulative pore volume for different paper materials; Figure S2. (a,b) The sensitivity curves of lens paper-based and rice paper-based capacitive pressure sensors after multiple pressure loads; Figure S3. (a) Schematic diagrams of the resistive strain sensor under tension (above) and without tension (below); (b) cross-sectional shape variation of the resistive strain sensor and the calculation formula for strain; Table S1. Comparison with research of pressure sensors prepared with cellulosic materials; Table S2. Comparison with research of strain sensors prepared with cellulosic materials. References [33,34,35,36,37,38,39,40,41] are cited in the Supplementary Materials.
