# Supplementary material for: Highly Sensitive Paper-Based Force Sensors with Natural Micro-Nanostructure Sensitive Element
Source: Nanomaterials (Basel). 2024 Feb 14;14(4):358. doi: 10.3390/nano14040358 (PMC10892271; doi:10.3390/nano14040358)
Supplement: Supplementary file 1 [file nanomaterials-14-00358-s001.zip › nanomaterials-2837244-supplementary.pdf]

Supplementary Materials

Highly Sensitive Paper-Based Force Sensors with Natural Micro-Nanostructure Sensitive Element

Haozhe Zhang [1,2,3], Yuyu Ren [4], Junwen Zhu [1,2,3], Yanshen Jia [1,2,3], Qiang Liu [1,2,3] and Xing Yang [1,2,3]*

[1] Department of Precision Instrument, Tsinghua University, Beijing 100084, China; zhanghz18@tsinghua.org.cn (H.Z.); zhujw21@mails.tsinghua.edu.cn (J.Z.); qiangliu@tsinghua.edu.cn (Q.L.); jys20@tsinghua.org.cn (Y.J.)

[2] Key Laboratory of Photonic Control Technology (Tsinghua University), Ministry of Education, Beijing 100084, China

[3] State Key Laboratory of Precision Space-time Information Sensing Technology, Beijing 100084, China

[4] School of Materials Science and Engineering, University of Science and Technology Beijing, Beijing 100083, China; 13519110246@163.com

* Correspondence: yangxing@tsinghua.edu.cn

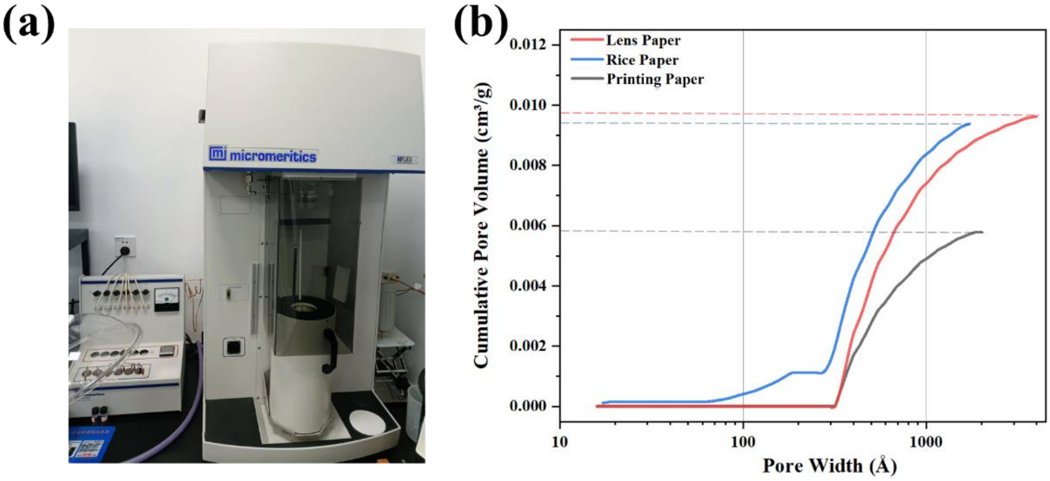

Figure S1. (**a**) BET experimental apparatus used for testing; (**b**) Comparison of cumulative pore volume for different paper materials.

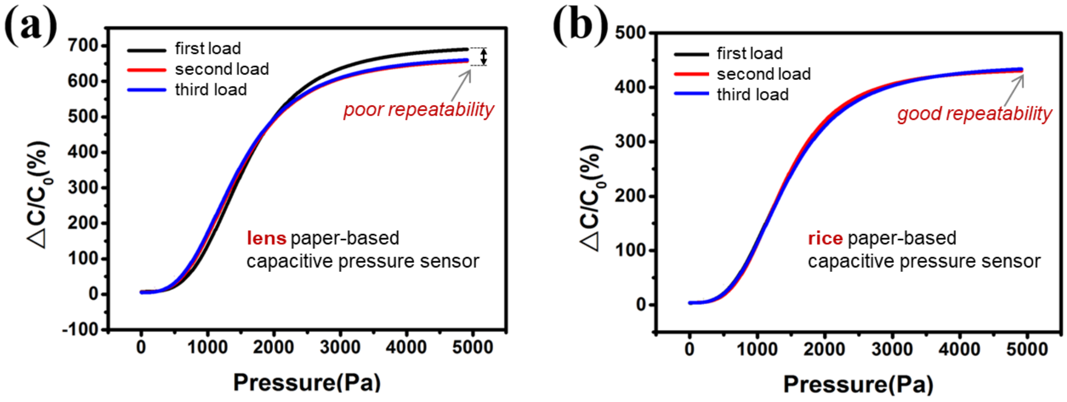

Figure S2. (**a**,**b**) The sensitivity curves of lens paper-based (left) and rice paper-based (right)

capacitive pressure sensors after multiple pressure loads.

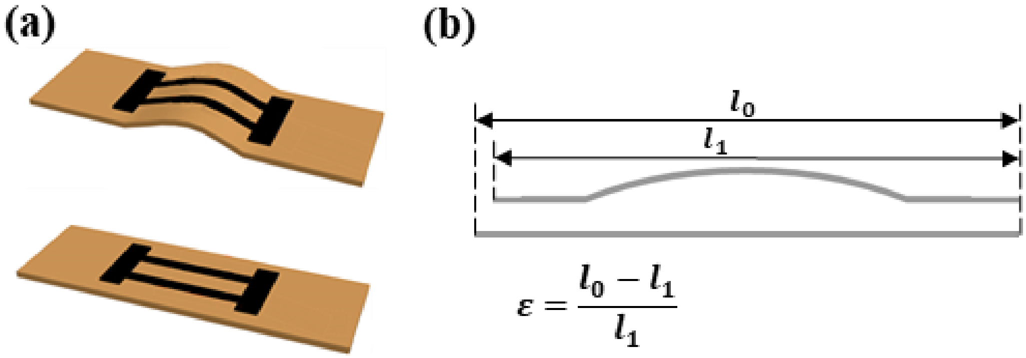

Figure S3. (**a**) Schematic diagrams of the resistive strain sensor under tension (above) and without tension (below); (**b**) Cross-sectional shape variation of the resistive strain sensor and the calculation formula for strain.

Table S1. Comparison with research of pressure sensors prepared with cellulosic materials

Research	Flexible substrate	Sensitive element	Sensitivity (kPa^{-1})	Response time (ms)
Ref.33	Bacterial cellulose	MXene	12.5	167
Ref.34	CNC	CNT/Graphene	0.25	
Ref.35	Cotton balls	Multiwalled carbon nanotube (MWCNT)	0.0197	20
Ref.36	Cellulose microcrystalline	Polypyrrole	58.9	
Ref.37	TEMPO CNF	Silver nanoparticle	9.5	
**Our work**	**Paper material**	**Paper material**	**1.623**	**240**

Table S2. Comparison with research of strain sensors prepared with cellulosic materials

Research	Flexible substrate	Sensitive element	Sensitivity	Response time (ms)
Ref.38	Bacterial cellulose	Multiwalled carbon nanotube (MWCNT)	21	390
Ref.39	Carboxymethyl cellulose	Na Fe^{+}3^{+}	4.02	260
Ref.40	Bacterial cellulose	MWCNT Carbon black	5.01	
Ref.41	CNF	Carbonized lignin		
**Our work**	**Paper material**	**Paper material**	**72**	**300**
